# Paternal Grandmother Age Affects the Strength of *Wolbachia*-Induced Cytoplasmic Incompatibility in Drosophila melanogaster

**DOI:** 10.1128/mBio.01879-19

**Published:** 2019-11-05

**Authors:** Emily M. Layton, Jungmin On, Jessamyn I. Perlmutter, Seth R. Bordenstein, J. Dylan Shropshire

**Affiliations:** a Department of Biological Sciences, Vanderbilt University, Nashville, Tennessee, USA; b Department of Pathology, Microbiology, and Immunology, Vanderbilt University Medical Center, Nashville, Tennessee, USA; c Vanderbilt Institute for Infection, Immunology and Inflammation, Vanderbilt University Medical Center, Nashville, Tennessee, USA; d Vanderbilt Microbiome Initiative, Vanderbilt University, Nashville, Tennessee, USA; Max Planck Institute for Marine Microbiology

**Keywords:** *Wolbachia*, *Drosophila melanogaster*, cytoplasmic incompatibility, maternal transmission

## Abstract

*Wolbachia* are obligate intracellular bacteria that are globally distributed in half of all arthropod species. As the most abundant maternally inherited microbe in animals, *Wolbachia* manipulate host reproduction via reproductive parasitism strategies, including cytoplasmic incompatibility (CI). CI manifests as embryonic death when *Wolbachia*-modified sperm fertilize uninfected eggs but not maternally infected eggs. Thus, CI can provide a relative fitness advantage to *Wolbachia*-infected females and drive the infection through a population. In the genetic model Drosophila melanogaster, the *Wolbachia* strain *w*Mel induces variable CI, making mechanistic studies in D. melanogaster cumbersome. Here, we demonstrate that sons of older paternal D. melanogaster grandmothers induce stronger CI than sons of younger paternal grandmothers, and we term this relationship the “paternal grandmother age effect” (PGAE). Moreover, the embryos and adult sons of older D. melanogaster grandmothers have higher *Wolbachia* densities, correlating with their ability to induce stronger CI. In addition, we report that *Wolbachia* density positively correlates with female age and decreases after mating, suggesting that females transmit *Wolbachia* loads that are proportional to their own titers. These findings reveal a transgenerational impact of age on *w*Mel-induced CI, elucidate *Wolbachia* density dynamics in D. melanogaster, and provide a methodological advance to studies aimed at understanding *w*Mel-induced CI in the D. melanogaster model.

## Abstract (precis)

Unidirectional cytoplasmic incompatibility (CI) results in a postfertilization incompatibility between *Wolbachia*-infected males and uninfected females. CI contributes to reproductive isolation between closely related species and is used in worldwide vector control programs to drastically lower arboviral vector population sizes or to replace populations that transmit arboviruses with those resistant to transmission. Despite decades of research on the factors that influence CI, penetrance is often variable under controlled laboratory conditions in various arthropods, suggesting that additional variables influence CI strength. Here, we demonstrate that paternal D. melanogaster grandmother age influences the strength of CI induced by their sons. Older D. melanogaster females have higher *Wolbachia* densities and produce offspring with higher *Wolbachia* densities that associate with stronger CI. This work reveals a multigenerational impact of age on CI and expands our understanding of host-*Wolbachia* interactions and the biology of CI induced by the *Wolbachia* strain infecting the most widely used arthropod model, D. melanogaster.

## INTRODUCTION

*Wolbachia* are obligate intracellular bacteria that infect 40% to 65% of arthropod species (1–3) and 37% of the members of the Onchocercidae family of filarial nematodes (4). These bacteria are maternally transmitted from ova to offspring (5) and often cause cytoplasmic incompatibility (CI) to selfishly increase their transmission through the matriline (6–10). CI manifests as embryonic death when *Wolbachia*-modified sperm fertilize uninfected eggs but not when they fertilize infected eggs (11–13). Thus, infected transmitting females have a fitness advantage relative to their uninfected counterparts that leads to the spread of *Wolbachia* through host populations (6–10). Additionally, since CI reduces gene flow between *Wolbachia*-infected and uninfected populations or populations with different *Wolbachia* strains, it is associated with reproductive isolation and incipient speciation (14, 15).

Global vector control efforts have successfully leveraged CI to either suppress native populations (16–19) or promote the spread of disease-resistant *Wolbachia* strains (20–22) specifically through release of mosquitoes transinfected with the *w*Mel *Wolbachia* strain of Drosophila melanogaster. *w*Mel's success in these efforts is partially due to the strong CI that it induces in mosquito hosts (23, 24); however, in the native host D. melanogaster, *w*Mel’s CI strength can range from an average of nearly 0% (no CI) to 100% (complete CI) (25–32). There are numerous factors reported to impact the penetrance of *w*Mel-induced CI: *Wolbachia* density in the testes (25, 33), expression level of the CI genes *cifA* and *cifB* (29, 34), male age (30), male mating rate (30, 35), time of male emergence (32), fly rearing density (32), and temperature (30). However, these factors are not independent, and they have likely hampered the researcher's ability to use the vast resources of D. melanogaster for the study of reproductive parasitism and endosymbiosis. For example, CI strength rapidly decreases with male age (30), which also cocorrelates with *cifA* and *cifB* gene expression (29) and *Wolbachia* density in the testes (33).

Despite control of male age, time of emergence, rearing density, and temperature, we continued to see various levels of CI strength in our laboratory, suggesting that additional factors are involved. This variation in phenotype makes *w*Mel in D. melanogaster difficult to study despite the fly’s extensive history as a powerful animal model. However, anecdotal observations in our laboratory suggested that stronger CI was induced in embryos when their infected paternal grandmothers were significantly aged before mating. Here, we used hatch rate analyses to formally test the hypothesis that paternal grandmother age influences the strength of CI induced by her sons. We also measured the effect of age and virginity on female *Wolbachia* titers and assessed whether females with higher *Wolbachia* titers deposited more *Wolbachia* into their progeny. Our results reveal a “paternal grandmother age effect” (PGAE) on CI strength, where older grandmothers produce males that induce stronger CI. We also characterize transgenerational *Wolbachia* density dynamics that correlate with CI penetrance. This work enhances our understanding of *Wolbachia*-host dynamics and provides methodological techniques of importance to studies of *w*Mel-induced CI in D. melanogaster.

## RESULTS

**FIG 1:**
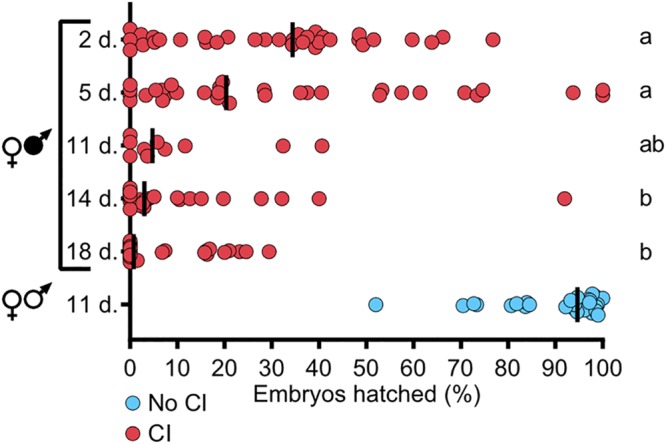
Paternal grandmother age effect impacts CI strength. Hatch rate assays were conducted with either uninfected y^1^w* males derived from uninfected females aged 11 days (d.) before mating or infected y^1^w* males derived from infected females aged 2, 5, 11, 14, or 18 days. *Wolbachia* infections are represented by filled sex symbols, and the age of the paternal grandmother is shown immediately to the left of the *y* axis. Each dot represents a replicate of offspring from single-pair matings. Vertical bars represent medians, and letters to the right indicate significant differences based on α = 0.05 calculated by a Kruskal-Wallis test followed by a Dunn’s multiple-comparison test performed between all CI crosses. All statistical values are presented in Table S1 in the supplemental material.

To test the hypothesis that D. melanogaster paternal grandmother age influences the strength of CI, we measured the percentage of surviving offspring produced by sons of differentially aged, infected y^1^w* grandmothers. CI strength increased with grandmother age when uninfected females were mated to infected sons of 2-, 5-, 11-, 14-, and 18-day-old grandmothers (Fig. 1). Sons of 2-day-old grandmothers produced statistically weaker CI than those of either 14-day-old (*P* = 0.0031) or 18-day-old (*P* = 0.0005) grandmothers, and the same was true for sons of 5-day-old grandmothers compared to those of either 14-day-old (*P* = 0.0095) or 18-day-old (*P* = 0.0018) grandmothers. Importantly, sons of 11-day-old uninfected grandmothers produced high hatch rates (Fig. 1), suggesting that the reduction in hatch rate in the remaining crosses was not associated with further aging of the flies. Together, these data suggest that CI is strongest in sons of older grandmothers (Fig. 1).

10.1128/mBio.01879-19.5 TABLE S1 *P* values associated with all statistical comparisons corresponding to the main and extended bodies of data presented in the figures. Download Table S1, DOCX file, 0.01 MB. Copyright © 2019 Layton et al. 2019 Layton et al. This content is distributed under the terms of the Creative Commons Attribution 4.0 International license.

Next, we tested whether the increase in embryonic death with D. melanogaster grandmother age indeed represented CI and not some other transgenerational embryonic defect. In accordance with prior results (Fig. 1), there was an overall trend indicating that older grandmothers produced sons that induced stronger CI. Indeed, sons derived from 2-day-old infected grandmothers induced statistically weaker CI than sons of 11-day-old (*P* = 0.0008) and 14-day-old (*P* = 0.0110) grandmothers (see Fig. S1 in the supplemental material). Sons of 11-day-old grandmothers produced a lower median hatch rate than sons of 14-day-old grandmothers; however, the differences were not statistically significant (*P* > 0.9999). As expected for CI rescue, high rates of embryonic hatching were observed when infected females were mated to sons of infected 2-, 5-, 11-, and 14-day-old grandmothers and the rates did not differ significantly between groups (Fig. S1; *P* = 0.3705). Together, these results suggest that the PGAE is not attributable to other transgenerational, age-associated defects.

10.1128/mBio.01879-19.1 FIG S1 Paternal D. melanogaster grandmother age does not impact hatching in non-CI crosses. Hatch rate assays were conducted with uninfected or infected y^1^w* males derived from females aged 2, 5, 11, or 14 days (d.) followed by mating crossed to either infected or uninfected y^1^w* females. Each dot represents a replicate of offspring from single-pair matings. *Wolbachia* infections are represented by filled sex symbols, and the age of the paternal grandmother is shown immediately to the left of the *y* axis. Vertical bars represent medians, and letters to the right indicate significant differences based on α = 0.05 calculated by a Kruskal-Wallis test followed by a Dunn’s multiple-comparison test performed between crosses (in brackets). All statistical values are presented in Table S1. Download FIG S1, TIF file, 0.2 MB. Copyright © 2019 Layton et al. 2019 Layton et al. This content is distributed under the terms of the Creative Commons Attribution 4.0 International license.

To test if the PGAE is specific to the y^1^w* strain, these experiments were repeated in a *nos*-GAL4-*tubulin* genetic background. The *nos-*GAL4-*tubulin* line was chosen because it was previously used to identify the *cifA* and *cifB* genes that underpin *w*Mel-induced CI (29). The 2-, 5-, and 11-day time points were selected because they had demonstrated the greatest differences in hatch rate in the previous experiments. As predicted, CI strength correlated with the age of paternal grandmothers when uninfected *nos*-GAL4-*tubulin* females were mated to infected sons of 2-, 5-, and 11-day-old *nos*-GAL4-*tubulin* grandmothers (Fig. S2). Sons of 11-day-old grandmothers induced significantly stronger CI than sons of 2-day-old grandmothers (*P* = 0.0033; Fig. S2), suggesting that the PGAE is not specific to y^1^w* flies. When sons of uninfected grandmothers aged 2, 5, or 11 days were mated to uninfected females, there were no statistically significant differences in hatching rates across all three groups (*P* = 0.3907; Fig. S2), indicating that the PGAE is CI associated in *nos*-GAL4-*tubulin* flies as seen with y^1^w* flies.

10.1128/mBio.01879-19.2 FIG S2 The paternal D. melanogaster grandmother age effect is not specific to y^1^w* flies. Hatch rates assays were conducted with uninfected or infected *nos*-GAL4-*tubulin* males derived from females aged 2, 5, or 11 days (d.) followed by mating crossed to uninfected *nos*-GAL4-*tubulin* females. Each dot represents a replicate of offspring from single-pair matings. *Wolbachia* infections are represented by filled sex symbols, and the age of the paternal grandmother is shown immediately to the left of the *y* axis. Vertical bars represent medians, and letters to the right indicate significant differences based on α = 0.05 calculated by a Kruskal-Wallis test followed by a Dunn’s multiple-comparison test performed between crosses (in brackets). All statistical values are presented in Table S1. Download FIG S2, TIF file, 0.2 MB. Copyright © 2019 Layton et al. 2019 Layton et al. This content is distributed under the terms of the Creative Commons Attribution 4.0 International license.

**FIG 2:**
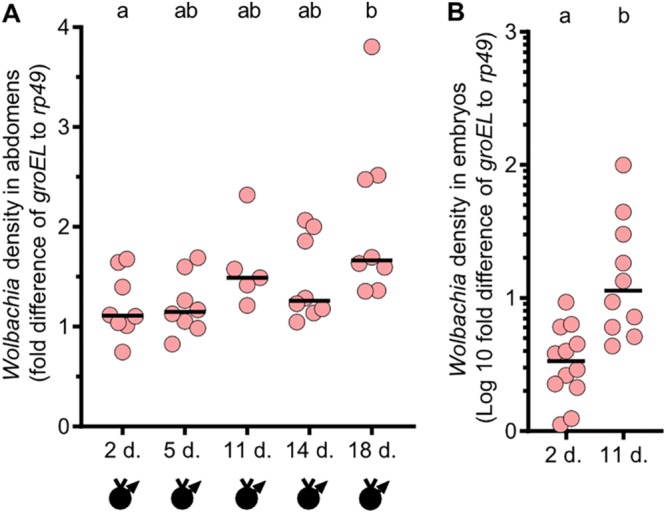
*Wolbachia* densities are highest in sons and embryos of older D. melanogaster grandmothers. (A) *Wolbachia* density assays were conducted with virgin females (indicated by a “v” above a sex symbol) and with infected y^1^w* males derived from grandmothers aged 2, 5, 11, 14, or 18 days (d.). *Wolbachia* infections are represented by filled sex symbols, and the age of the grandmother is shown immediately below the *x* axis. The samples analyzed were from abdomens of siblings of fathers corresponding to the hatch rate data in Fig. 1. (B) *Wolbachia* density assays were conducted with pools of 50 1-to-2-h-old embryos collected from 2-and-11-day-old grandmothers. The sex of the embryos was unknown since it cannot be determined visually. *Wolbachia* titers were lower in adults, requiring a standard linear scale (A), but higher in embryos, requiring a common logarithmic scale (B). Each dot represents the average of results from triplicate technical replicates for panel A and duplicates for panel B. Horizontal bars indicate medians, and the letters above the bars indicate significant differences based on α = 0.05 calculated by Kruskal-Wallis test followed by a Dunn’s multiple-comparison test performed between all groups (A) or by a Mann Whitney *U* test (B). All statistical values are presented in Table S1. Fold differences in *Wolbachia* densities (*groEL*) relative to D. melanogaster reference gene *rp49* were determined with 2^−ΔΔ^*^CT^*.

Since *Wolbachia* densities are positively associated with CI strength (25, 36–38), we then tested the hypothesis that infected sons derived from older D. melanogaster grandmothers have higher *Wolbachia* densities than infected sons from younger grandmothers. We did so by measuring the abundance of the single-copy *Wolbachia groEL* gene relative to that of the *Drosophila rp49* housekeeping gene. Abdomen samples were taken from virgin male siblings of those used in the hatch rate experiment represented in Fig. 1. As predicted, *Wolbachia* densities in male abdomens positively correlated with paternal grandmother age, and sons of 18-day-old grandmothers had significantly higher *Wolbachia* densities than sons of 2-day-old grandmothers (*P* = 0.0450) (Fig. 2A). However, no significant differences were observed between sons of 5-, 11-, or 14-day-old grandmothers relative to any other group, presumably due to the variable penetrance of CI, low sample sizes, or biological reasons proposed in the Discussion. Taken together, these data suggest that older grandmothers produced sons with higher *Wolbachia* titers, which allowed the sons to induce stronger CI, though this density effect was weak relative to the effect that we see for CI.

Next, we tested the hypothesis that embryos from older D. melanogaster grandmothers had higher *Wolbachia* titers than those from younger grandmothers. *Wolbachia* densities were measured in 0-to-1-h-old embryos produced by both 2-day-old and 11-day-old grandmothers (Fig. 2B). The 2-day and 11-day time points were chosen because they exhibited the greatest differences in CI strength over the shortest time interval. Here, embryos produced by 11-day-old grandmothers had significantly higher *Wolbachia* densities than embryos from 2-day-old grandmothers (*P* = 0.0006) (Fig. 2B). Thus, these data indicate that older females produce embryos with higher *Wolbachia* titers.

**FIG 3:**
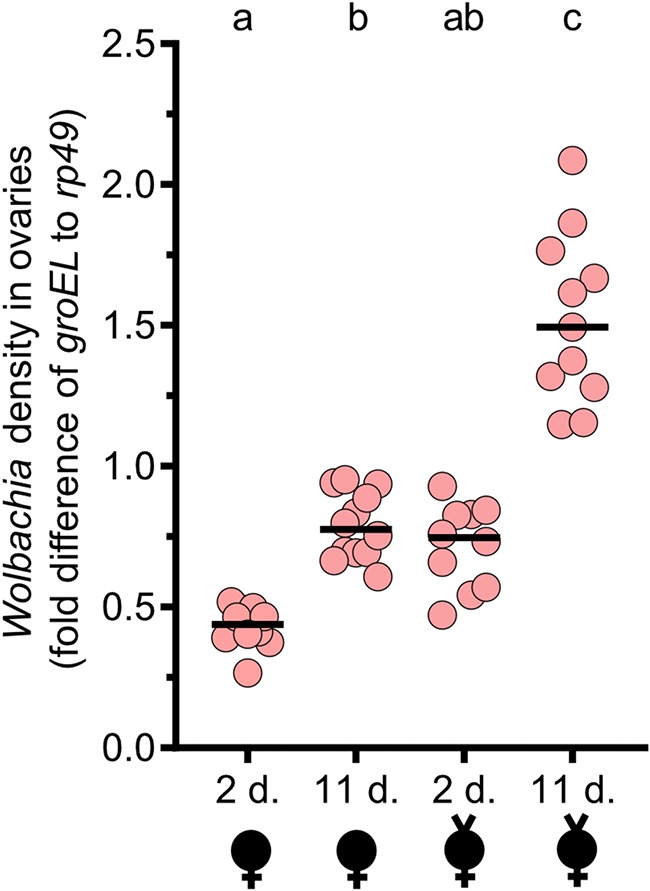
*Wolbachia* densities increase with female age in ovaries and decrease after mating. *Wolbachia* density assays were conducted with pools of 4 ovaries from virgin females (indicated by a “v” above a sex symbol) and nonvirgin females aged 2 or 11 days (d.). *Wolbachia* infections are represented by filled sex symbols, and the age of the sample is shown immediately below the *x* axis. Virgin and nonvirgin females were siblings. The nonvirgin females produced the embryos whose results are shown in Fig. 2B. Nonvirgin females were allowed to mate and lay for 48 h before ovary dissections. Nonvirgin and virgin females were incubated for that same period of time and dissected in parallel. Each dot represents the average of duplicate values. Horizontal bars indicate medians, and the letters above the bars indicate significant differences based on α = 0.05 calculated by a Kruskal-Wallis test followed by a Dunn’s multiple-comparison test performed between all groups. All statistical values are presented in Table S1. Fold differences in *Wolbachia* density (*groEL*) relative to D. melanogaster reference gene *rp49* were determined with 2^−ΔΔ^*^CT^*.

Finally, this led to the hypothesis that older D. melanogaster grandmothers have higher *Wolbachia* densities than younger grandmothers and that they transfer more *Wolbachia* to their offspring. Supporting this hypothesis, *Wolbachia* densities were significantly higher in the ovaries of 11-day-old virgin females than in those of 2-day-old virgin females (*P* = 0.0045) (Fig. 3). Additionally, we predicted that *Wolbachia* densities would decrease in ovaries after egg-laying if grandmothers loaded *Wolbachia* into their offspring. As such, we measured *Wolbachia* densities in ovaries of mated grandmothers that laid eggs in the embryo density study described previously. We found that ovaries from mated 11-day-old females had significantly less *Wolbachia* than virgin 11-day-old females (*P* = 0.0240) (Fig. 3). Likewise, mated 2-day-old females had lower *Wolbachia* titers than virgin 2-day-old females, though the differences were not significant (*P* = 0.0882) (Fig. 3). Despite the overall decrease in relative *Wolbachia* densities after mating, ovaries from 11-day-old mated grandmothers had significantly higher densities than ovaries from 2-day-old mated grandmothers (*P* = 0.0087). Importantly, threshold cycle (*C_T_*) values remained consistent across age and virginity states for the *Drosophila rp49* gene, suggesting that changes in the *Wolbachia groEL* gene, rather than in *rp49* copy number, were responsible for the density dynamics that we report here (Fig. S3).

10.1128/mBio.01879-19.3 FIG S3 *rp49 C_T_* values remain consistent across age and virginity states, whereas *groEL* fluctuates. *C_T_* values are from the *Wolbachia* density analysis described in the Fig. 3 legend. *Wolbachia* density assays were conducted with pools of 4 ovaries from virgin females (indicated by a “v” above a sex symbol) and nonvirgin females aged 2 or 11 days (d.). *Wolbachia* infections are represented by filled sex symbols, and the age of the sample is shown to the left of the sex symbol. Virgin and nonvirgin females were siblings. The nonvirgin females produced the embryos represented in Fig. 2B. Nonvirgin females were allowed to mate and lay for 48 h before ovary dissections were performed. Nonvirgin and virgin females were incubated for the same period of time and dissected in parallel. Each dot represents the average of duplicate values. Vertical bars indicate medians, and the letters above the bars indicate significant differences based on α = 0.05 calculated by a Kruskal-Wallis test followed by a Dunn*’*s multiple-comparison test performed between all virgin females and mated females (separated by a horizontal dotted line). All statistical values are presented in Table S1. Download FIG S3, TIF file, 0.2 MB. Copyright © 2019 Layton et al. 2019 Layton et al. This content is distributed under the terms of the Creative Commons Attribution 4.0 International license.

Similar results can be observed in measuring *Wolbachia* densities in abdomens instead of in ovaries (Fig. S4). Measuring abdominal titers, 11-day-old virgin females had statistically higher *Wolbachia* densities than 2-day-old virgin females (*P* < 0.0001). There was a detectable trend indicating that the mated females had less *Wolbachia*, though neither mated 11-day-old females (*P* = 0.2291) nor mated 2-day-old females (*P* > 0.9999) had titers significantly different from those of their virgin counterparts (Fig. S4). The titers in 11-day-old and 2-day-old mated females were not significantly different (*P* > 0.9999). Taken together, these data suggest that females accumulate *Wolbachia* as they age, that older females transfer more *Wolbachia* to their offspring, and that sons of older females induce stronger CI. Moreover, laying eggs appears to quickly reduce the amount of *Wolbachia* contained in the ovaries, suggesting that the PGAE is strongest soon after initial mating.

10.1128/mBio.01879-19.4 FIG S4 *Wolbachia* densities increase with female age in abdomens and decrease after mating. *Wolbachia* density assays were conducted with single abdomens from virgin females (indicated by a “v” above a sex symbol) and nonvirgin females aged 2 or 11 days (d.). *Wolbachia* infections are represented by filled sex symbols, and the age of the sample is shown immediately below the *x* axis. Virgin and nonvirgin females were siblings. The nonvirgin females produced the fathers corresponding to the hatch rate data in Fig. 1 and the males whose data are shown in Fig. 2A. Nonvirgin females were allowed to mate and lay for 48 h before abdomen dissections were performed. Nonvirgin and virgin females were incubated for that same period of time and dissected in parallel. Each dot represents the average of triplicate values. Horizontal bars indicate medians, and the letters above the bars indicate significant differences based on α = 0.05 calculated by a Kruskal-Wallis test followed by a Dunn’s multiple-comparison test performed between all groups. All statistical values are presented in Table S1. Fold differences in *Wolbachia* density (*groEL*) relative to D. melanogaster reference gene *rp49* were determined with 2^−ΔΔ^*^CT^*. Download FIG S4, TIF file, 0.2 MB. Copyright © 2019 Layton et al. 2019 Layton et al. This content is distributed under the terms of the Creative Commons Attribution 4.0 International license.

## DISCUSSION

D. melanogaster is a valued model system in studies of *Wolbachia*-host interactions due to its genetic tractability and the importance of its native *Wolbachia* strain, *w*Mel, in vector control (39). However, the study of *w*Mel-induced CI in D. melanogaster is inhibited by its variable penetrance, ranging from nearly complete embryonic death to none at all (26–32). Some phenotypic variation persists despite control of known variables of CI strength, leading to the hypothesis that as-yet-unknown factors contribute to CI variability. Anecdotal observations in our laboratory suggested that stronger CI may be induced by offspring of older virgin females, leading to the formal hypothesis that variation in CI penetrance is partly controlled by a paternal grandmother age effect (PGAE).

Here, we report evidence in support of the PGAE, namely, that sons of older D. melanogaster grandmothers induce stronger CI than sons of younger grandmothers. Paternal grandmother age did not influence the ability of CI to be rescued, suggesting that no other age-associated transgenerational deficiencies contributed to the increased embryonic death. Additionally, we found that embryos of older grandmothers had higher *Wolbachia* densities than the offspring of younger grandmothers. Likewise, older virgin females had more *Wolbachia* than younger virgin females. As such, the data support a model whereby PGAE is caused by an accumulation of *Wolbachia* in a virgin as she ages, leading to an increase in levels of *Wolbachia* passed on to her sons, who induce stronger CI in their offspring than sons of younger grandmothers.

In this study, we measured *Wolbachia* densities by comparing the number of *Wolbachia groEL* gene copies to the number of *Drosophila rp49* gene copies. Note that we cannot make direct claims about the density of *Wolbachia* per host cell based on these analyses, since doing so would assume that the number of host cells and host ploidy remain constant. Recent work has highlighted that a protein-enriched diet can influence relative estimates of *Wolbachia* density analysis in D. melanogaster by increasing ovary size and *rp49* copy number (40). While age and mating state may be hypothesized to influence *rp49* copy number, *rp49 C_T_* values remained constant across female age and mating states whereas *groEL C_T_* values changed (see Fig. S3 in the supplemental material). These data suggest that despite possible fluctuations in *rp49* copy number across cell types within ovaries, the average *rp49* copy number remains consistent across the extracted tissue samples. As such, we conclude that changes in *Wolbachia groEL* copy number, not *rp49* copy number, underpin the results. However, future work will be necessary to describe how these density estimates explicitly relate to *Wolbachia* titers per host cell and across cell types in these tissues.

In addition to the PGAE, CI variation has previously been attributed to a “younger-brother effect” where the slowest-developing males, from a clutch of embryos within the 0-to-5-h age range, induced the weakest CI (32). If embryo deposition order correlates with maturation rate, then the younger-brother effect is at least in part explained by our findings that (i) *Wolbachia* densities in ovaries quickly decrease after mating and egg laying, (ii) the *Wolbachia* density in embryos correlates with ovary densities, and (iii) sons from eggs laid by mothers with lower *Wolbachia* densities induce weaker CI. As such, when a D. melanogaster female lays eggs, the amount of *Wolbachia* in her ovaries may be sequentially depleted after each embryo is produced. Thus, younger brothers that take longer to develop may receive fewer *Wolbachia* and then induce a weaker CI than their older counterparts that originally had received more *Wolbachia*. Therefore, the dynamics of the interaction that we observed between CI induction and *Wolbachia* densities across generations may explain the younger-brother effect, although this remains to be precisely established in future research.

Additionally, this paper adds to a growing body of literature reporting an influence of female insect age on *Wolbachia* densities. Indeed, older females harbor higher *Wolbachia* titers in *w*AlbA- and *w*AlbB-infected Aedes albopictus (41, 42), *w*VulC-infected Armadillidium vulgare (43), and *w*Stri-infected Laodelphax striatellus (44). The relationship between paternal grandmother age and the strength of *w*Mel-induced CI was explored once before; however, no relationship was found (32). Crucially, the virginity status of the grandmothers differs between the cited study and the one presented here and may in part explain the discrepancy. Our study maintained the virginity of all grandmothers as they aged, and grandmothers were allowed only 24 h of mating prior to egg deposition for hatch rate analysis. In contrast, the grandmothers in the prior study remained virgin until 3 days old and were then allowed to continuously mate until they were 11 days old, and the CI levels from sons produced at each of the two time points were compared (32). Our results suggest that mating has a detectable impact on *Wolbachia* densities and may explain why the PGAE was not observed in the earlier study. Additionally, we predict that the PGAE most strongly applies to aged virgins, since mating significantly reduced *Wolbachia* densities in our study.

The depletion of *Wolbachia* found in females following egg laying supports the hypothesis that the PGAE is caused by an effect of maternal loading of *Wolbachia* into her sons. However, the source of that loading is still unclear. In D. melanogaster, the following four sources of *Wolbachia* transfer to progeny are known: bacteriocyte-like cells (BLCs), germ line stem cells (GSCs), the somatic stem cell niche (SSCN), and late-stage oogenesis (5, 38, 45–48). BLCs found at the tip of the ovarioles are densely packed with *Wolbachia* and are predicted to transfer *Wolbachia* to GSCs (47). When a GSC asymmetrically undergoes mitosis (49, 50), its population of *Wolbachia* is divided between two daughter cells, one of which is an identical GSC that remains in the ovaries and the other a differentiating cytoblast that develops into the egg (5). Therefore, it is possible that the levels of *Wolbachia* allocated to the daughter cytoblast (and thus the offspring) are proportional to the densities in the parent GSC or the surrounding BLCs. Additionally, as the cytoblast develops into a germ line cyst, it comes into contact with the highly infected SSCN, acquiring additional *Wolbachia* (45, 46, 48). Finally, while *Wolbachia* replication in the oocyte occurs primarily at the beginning of oogenesis in *w*Mel-infected D. melanogaster and halts at the onset of vitellogenesis, it can resume at a lower rate before egg laying in late-stage oogenesis (38). As such, prolonged retention of eggs in aged virgins may lead to an accumulation of *Wolbachia* in these developed oocytes. We hypothesize that *Wolbachia* replicate in the BLCs, GSCs, SSCNs, or late-stage oocytes as a mother ages, resulting in eggs with relatively high titers. Since eggs account for the greatest proportion of *Wolbachia* cells in the ovaries, this hypothesis could explain why titers are depleted after mating and egg laying.

Intriguingly, differences in CI strength more closely correlated with *Wolbachia* densities in embryos than with densities in adult males. CI is hypothesized to be caused by *cif* gene modifications of sperm-associated host products (51–58) or to be a consequence of loading of toxins into the sperm (52, 53, 59, 60); however, *Wolbachia* are stripped from the sperm during individualization (37, 61, 62). Therefore, *Wolbachia* titers are likely more important during a specific stage of spermatogenesis than at the time of CI induction. In D. melanogaster, spermatogenesis is a continuous process lasting approximately 11 days (63). As such, there may be a lag of several days between the time that sperm are subjected to the actions of *cifA* and *cifB* gene products and the time of CI induction. Spermatogenesis begins during larval development (63) and continues throughout the adult life span (64), though the first batches of mature sperm are produced soon after adult hatching (65). Since the males in our study were mated shortly after adult hatching, the majority of their sperm would have started spermatogenesis at a time closer to embryonic deposition than adult hatching, which may explain why CI strength correlates better with *Wolbachia* densities in embryos than in adult males. Additionally, spermatogenesis may incorporate and eliminate *Wolbachia* faster than they can multiply, resulting in the reduction and equalization of titers in adults (37). This may explain why some studies, including studies analyzing the younger-brother effect, found that CI strength did not always correlate with *Wolbachia* densities in adults (25, 32, 66). As such, we predict that the PGAE is the result of the presence of high *Wolbachia* densities during a critical time point in spermatogenesis when CI-defining changes occur, which may become the subject of future research.

It remains unclear if the association between female age and *Wolbachia* densities would be the case in wild populations. Since wild D. melanogaster females are estimated to mate, on average, every 27 h (67), it would seem unlikely that the *Wolbachia* accumulation reported here would occur in nature. However, infection status has been reported to influence mate choice behaviors in numerous animals, including D. subquinaria, D. paulistorum, Nasonia vitripennis, and Tetranychus urticae (68–71). For example, male mating rate affects CI strength (30), so *w*Mel-infected males mate more frequently to reduce the impact of CI strength and therefore improve their lifetime reproductive success (35). Additionally, females infected with *Wolbachia* have a higher reproductive fitness when their daughters can sufficiently rescue CI and when their sons induce weak CI. Thus, it is plausible that the latency to copulation could be either lengthened in instances where a higher *Wolbachia* titer would be preferable (rescue efficiency) or, conversely, shortened in populations where a lower density is preferred (weakened CI). While it is unlikely that a fly in nature will remain virgin for as long as reported in this study, it is notable that CI strength increased substantially with every time point measured. As such, even small changes in mating latency may influence CI strength sufficiently to change the rate of spread through a population. Field studies measuring the latency toward copulation in sites with different infection rates would help determine if insects can modulate their mating latency, and thus *Wolbachia* titers, to increase their fitness and the fitness of their offspring.

While this work reports a PGAE for *w*Mel in D. melanogaster, it is unknown if these dynamics occur for *w*Mel in mosquito hosts. In *w*Mel-infected Aedes aegypti mosquitoes, CI is consistently strong (23, 24). However, some factors such as *Wolbachia* densities and temperature were shown previously to correlate with CI penetrance (72). It is possible that other as-yet-unstudied factors in mosquitoes, such as the PGAE, can contribute to changes in CI strength. Since strong CI is crucial for rapid spread of *w*Mel-infected mosquitoes through populations for successful vector control applications (73), understanding the factors that contribute to variation in CI strength would further inform the efficacy of population replacement and rearing strategies. Moreover, comparative studies exploring *w*Mel-induced CI in D. melanogaster and A. aegypti could clarify the *Wolbachia*-host dynamics that govern the penetrance of CI.

Finally, there is a striking range of CI penetrance across *Wolbachia* and hosts, and more work is necessary to determine if the PGAE applies to other CI or reproductive parasite systems. For example, *w*Ri in D. simulans consistently induces strong CI (7, 10, 74) and *w*Yak and *w*Tei in the D. yakuba clade cause weak and variable levels of CI similar to those seen with *w*Mel (75). Intriguingly, *w*Mel and *w*Tei were initially thought not to cause CI until factors such as male age and host genotype were found to have a significant impact on CI strength (30, 75–79). Since it is clear that some *Wolbachia* cause CI only under strictly limited conditions, it remains possible that other weak-CI-inducing *Wolbachia* are mislabeled as non-CI strains because factors such as the PGAE had not been controlled for during initial testing. Indeed, while this work presents the first reported case of transgenerational *Wolbachia* titers influencing CI, it is not the first case of transgenerational *Wolbachia* titers influencing reproductive parasitism. In D. innubila, male-killing *Wolbachia* frequently kill all male offspring, but females with lower *Wolbachia* titers are known to produce some viable sons (80). The surviving female offspring inherit lower-than-average *Wolbachia* titers, leading to a greater-than-average chance that those infected females would also produce sons (80). Together, our results and those in D. innubila suggest that a transgenerational effect of titers may be common and consequential with respect to the expression of reproductive parasitism traits.

In conclusion, we characterize *Wolbachia* density dynamics in females in relation to age and mating, and we link a transgenerational influence of grandmother age to CI penetrance. This work highlights the importance of controlling grandparent age in future studies of *w*Mel-induced CI in D. melanogaster and has implications for laboratory experiments where precise control over levels of CI would be valuable for dissecting the genetic and functional basis of CI. Additionally, it expands our understanding of *Wolbachia*-host interactions in relation to CI penetrance and titer dynamics and should motivate additional studies exploring these interactions in *w*Mel-infected mosquitoes.

## MATERIALS AND METHODS

### Fly strains and maintenance.

The following D. melanogaster strains were used in this study: *wMel-*infected and uninfected variants of y^1^w* (BDSC 1495) and *nos*-GAL4-*tubulin* (BDSC 4442). Uninfected lines were generated through three generations of tetracycline treatment as previously described (29). All stocks were reared on 50 ml of a standard medium containing cornmeal, molasses, and yeast and were maintained at 25°C with a 12-h/12-h light:dark cycle and at 70% relative humidity (RH). All virgin flies were collected using CO_2_ anesthetization per standard procedures. Briefly, virgin flies were collected in the morning based on the presence of a meconium, bottles were subsequently cleared of adult flies, and flies collected in the evening were assumed virgin due to the standard time of latency until mating. All virgin flies were kept at room temperature prior to experimentation.

### Hatch rate assays.

Hatch rate assays were used to assess the impact of D. melanogaster paternal grandmother age on the strength of CI induced by their sons. We conducted 3 variant hatch rate assays to test (i) whether paternal grandmother age influences CI hatch rates, (ii) whether this effect is specific to the y^1^w* genetic background, and (iii) whether the transgenerational impact of age on hatching is indeed caused by CI.

10.1128/mBio.01879-19.7 DATA SET S1 Source data for hatch rate and qPCR assays. Download Data Set S1, XLSX file, 0.7 MB. Copyright © 2019 Layton et al. 2019 Layton et al. This content is distributed under the terms of the Creative Commons Attribution 4.0 International license.

First, we assessed if D. melanogaster paternal grandmother age influences CI hatch rates in the y^1^w* genetic background. Paternal y^1^w* grandmothers were collected as virgins and allowed to reach 2, 5, 11, 14, or 18 days of age before mating in parallel with paternal grandfathers aged 0 to 2 days. Paternal grandparents from each age cohort were crossed in single-pair matings in standard vials of media. Since rearing density influences CI strength (32), paternal grandparents were allowed 24 h to mate and to deposit eggs before the grandfathers were discarded and the grandmothers were flash frozen and stored at –80°C for *Wolbachia* titer analysis. To control for the younger-brother effect and the effect of male age on the strength of CI (30, 32), the earliest eclosing fathers were collected as virgins and left to age 1 day at room temperature before being used in hatch rate assays.

Maternal y^1^w* grandparents were crossed in standard medium bottles and allowed to mate for 4 days before flies were cleared, as described above for the paternal grandparents. Mothers were collected as virgins and allowed to reach 6 to 8 days of age at room temperature to maximize fertility (81).

Parental y^1^w* mating pairs were placed in 8-oz *Drosophila* stock bottles (Genesee Scientific) with a grape juice agar plate covered in yeast affixed to the top to collect embryos for hatch rate analysis as previously described (29, 34). Parents were allowed two back-to-back 24-h mating and laying periods, each with separate freshly yeasted grape juice agar plates. The plates from the first mating period were discarded due to the typically low levels of egg laying in the first 24 h. The embryos from the second mating period were immediately counted after 24 h of additional laying. Embryos were then incubated for 30 h at 25°C to allow time to hatch. The unhatched embryos were counted, and the percentage of embryonic hatching was determined by dividing the number of unhatched embryos by the total number of embryos laid during the second mating period.

To minimize the effect of female fecundity on embryo viability (81), any plate with fewer than 25 embryos was excluded. We measured the hatch rates of offspring produced by two sons of each paternal grandmother. If both sons from the same family produced 25 or more embryos, one was randomly selected and used in analysis.

Next, to assess if the PGAE was specific to the y^1^w* genetic background, a separate hatch rate assay was conducted using *nos*-GAL4-*tubulin*-infected and uninfected flies. This experiment was conducted similarly to the hatch rate experiment described above, with the following adjustments: age and virginity of paternal grandfathers were not controlled. Paternal *nos*-GAL4-*tubulin*-infected and uninfected grandmothers were collected as virgins and allowed to reach 2, 5, or 11 days of age before they were allowed to mate in standard medium bottles, and these bottles were cleared of flies after 4 days of laying to control rearing density (32).

Finally, to determine if the PGAE was in fact due to *Wolbachia* and not to other forms of inviability induced by a transgenerational impact on age, we conducted compatible rescue crosses with males derived from 2-, 5-, 11-, or 14-day-old females. This experiment was conducted similarly to the hatch rate experiment described above, with the following adjustments: both infected and uninfected males were produced from virgin females aged 2, 4, 11, or 14 days; the uninfected males were mated to uninfected females; and infected males were mated to infected females. Paternal grandparents were paired in 8-oz *Drosophila* stock bottles (Genesee Scientific) with a grape juice agar plate (29) covered in yeast affixed to the top for a 24-h mating and laying period, and then grandparents were collected from the bottles. The plates were maintained for 24 h, and then 20 of the largest larvae were transferred from each plate to a standard medium vial to control rearing density (32).

### *Wolbachia* titer assays.

To assess the relationship between the PGAE and *Wolbachia* titers, the following tissues were collected: ovaries, female abdomens, embryos, and male abdomens. Since the low biomass of *Drosophila* testes requires them to be pooled, abdomens were used instead of testes so that samples could be taken directly from the males used in hatch rate assays. To test if virginity and age impact female *Wolbachia* titers, virgin and nonvirgin females 2 and 11 days of age were reared in parallel, ovaries were dissected in phosphate-buffered saline (PBS), and samples were frozen in liquid nitrogen followed by storage at –80°C. Samples consisted of 4 pairs of ovaries. Nonvirgin females were mated in cohorts of 60 females to 12 males, provided grape juice plates, and allowed 48 h to mate and lay eggs before dissection. Additionally, full bodies from 2-or-11-day-old paternal grandmothers from a hatch rate assay were collected alongside virgin paternal grandaunts (siblings to the paternal grandmothers), frozen in liquid nitrogen, and stored at –80°C. To determine if embryos derived from older females had higher *Wolbachia* titers, 0-to-1-h-old embryos were collected from grape plates in batches of 50, frozen in liquid nitrogen, and stored at –80°C. Finally, to assess whether the sons of aged paternal grandmothers had higher *Wolbachia* titers, full bodies from virgin uncles (siblings of fathers used in a hatch rate assay) derived from 2-, 5-, 11-, 14-, or 18-day-old grandmothers were collected and aged 48 h at room temperature in a standard medium vial. *Wolbachia* titers were measured in virgin uncles rather than the fathers used in the hatch rate assay because of the relationship between CI strength and male mating rate (35).

Upon removal from –80°C conditions, abdomens were immediately dissected from full-body tissues, homogenized in liquid nitrogen, and mixed with 40 μl ice-cold RNase-free PBS. Each sample was split, and 30% (12 μl) was flash frozen and stored at –80°C for DNA extractions. The DNA was extracted from all tissue types using a Gentra PureGene tissue kit (Qiagen). Forty cycles of quantitative PCR (qPCR) were performed using *rp49* and *groEL* primers (Table S2) for all DNA samples as well as positive controls (infected DNA), negative controls (uninfected DNA), no-reverse-transcription controls (RNA), and no-tissue controls (water). Male and female abdomen samples were tested in triplicate and ovaries and embryos in duplicate under the following qPCR conditions: 50°C for 10 min; 95°C for 5 min; 40 cycles of 95°C for 10 s and 55°C for 30 s; and 95°C for 30 s. Samples were excluded from analysis if the standard deviation of results of comparisons between replicates was >0.3. Fold difference between *Wolbachia* (*groEL*) density and that of the D. melanogaster *rp49* reference gene was determined with 2^−ΔΔ^*^CT^*.

10.1128/mBio.01879-19.6 TABLE S2 Primers used for qPCR. Download Table S2, DOCX file, 0.01 MB. Copyright © 2019 Layton et al. 2019 Layton et al. This content is distributed under the terms of the Creative Commons Attribution 4.0 International license.

### Statistical analyses.

All statistical analyses were conducted using GraphPad Prism 7. *Wolbachia* titers of embryos were analyzed using a Mann-Whitney *U* test. All other data (including data from hatch rate assays and ovary *Wolbachia* titer comparisons) were analyzed using the Kruskal-Wallis test followed by a Dunn’s multiple-comparison test. Figures were created in GraphPad Prism 7 and 8. All data used in these analyses have been made publicly available (see Data Set S1 in the supplemental material).

## References

[1] Hilgenboecker K, Hammerstein P, Schlattmann P, Telschow A, Werren JH 2008 How many species are infected with *Wolbachia*? – a statistical analysis of current data: *Wolbachia* infection rates. FEMS Microbiol Lett 281:215–220. doi:10.1111/j.1574-6968.2008.01110.x. 18312577 PMC2327208

[2] Weinert LA, Araujo-Jnr EV, Ahmed MZ, Welch JJ 2015 The incidence of bacterial endosymbionts in terrestrial arthropods. Proc Biol Sci 282:20150249. doi:10.1098/rspb.2015.0249. 25904667 PMC4424649

[3] Zug R, Hammerstein P 2012 Still a host of hosts for *Wolbachia*: analysis of recent data suggests that 40% of terrestrial arthropod species are infected. PLoS One 7:e38544. doi:10.1371/journal.pone.0038544. 22685581 PMC3369835

[4] Ferri E, Bain O, Barbuto M, Martin C, Lo N, Uni S, Landmann F, Baccei SG, Guerrero R, de Souza Lima S, Bandi C, Wanji S, Diagne M, Casiraghi M 2011 New insights into the evolution of *Wolbachia* infections in filarial nematodes inferred from a large range of screened species. PLoS One 6:e20843. doi:10.1371/journal.pone.0020843. 21731626 PMC3120775

[5] Serbus LR, Casper-Lindley C, Landmann F, Sullivan W 2008 The genetics and cell biology of *Wolbachia*-host interactions. Annu Rev Genet 42:683–707. doi:10.1146/annurev.genet.41.110306.130354. 18713031

[6] Hancock PA, Sinkins SP, Godfray HCJ 2011 Population dynamic models of the spread of *Wolbachia*. Am Nat 177:323–333. doi:10.1086/658121. 21460541

[7] Hoffmann AA, Turelli M, Harshman LG 1990 Factors affecting the distribution of cytoplasmic incompatibility in *Drosophila simulans*. Genetics 126:933–948. 2076821 10.1093/genetics/126.4.933 PMC1204290

[8] Jansen VAA, Turelli M, Godfray HCJ 2008 Stochastic spread of *Wolbachia*. Proc Biol Sci 275:2769–2776. doi:10.1098/rspb.2008.0914. 18755670 PMC2605827

[9] Turelli M 1994 Evolution of incompatibility-inducing microbes and their hosts. Evolution 48:1500–1513. doi:10.1111/j.1558-5646.1994.tb02192.x. 28568404

[10] Turelli M, Cooper BS, Richardson KM, Ginsberg PS, Peckenpaugh B, Antelope CX, Kim KJ, May MR, Abrieux A, Wilson DA, Bronski MJ, Moore BR, Gao J-J, Eisen MB, Chiu JC, Conner WR, Hoffmann AA 2018 Rapid global spread of *w*Ri-like *Wolbachia* across multiple *Drosophila*. Curr Biol 28:963–971.e8. doi:10.1016/j.cub.2018.02.015. 29526588 PMC5882237

[11] LePage D, Bordenstein SR 2013 *Wolbachia*: can we save lives with a great pandemic? Trends Parasitol 29:385–393. doi:10.1016/j.pt.2013.06.003. 23845310 PMC3775348

[12] Taylor MJ, Bordenstein SR, Slatko B 2018 Microbe profile: *Wolbachia*: a sex selector, a viral protector and a target to treat filarial nematodes. Microbiology 164:1345–1347. doi:10.1099/mic.0.000724. 30311871 PMC7008210

[13] Yen JH, Barr AR 1973 The etiological agent of cytoplasmic incompatibility in *Culex pipiens*. J Invertebr Pathol 22:242–250. doi:10.1016/0022-2011(73)90141-9. 4206296

[14] Bordenstein SR, O'Hara FP, Werren JH 2001 *Wolbachia*-induced incompatibility precedes other hybrid incompatibilities in *Nasonia*. Nature 409:707. doi:10.1038/35055543. 11217858

[15] Brucker RM, Bordenstein SR 2012 Speciation by symbiosis. Trends Ecol Evol 27:443–451. doi:10.1016/j.tree.2012.03.011. 22541872

[16] Dobson SL, Fox CW, Jiggins FM 2002 The effect of *Wolbachia*-induced cytoplasmic incompatibility on host population size in natural and manipulated systems. Proc Biol Sci 269:437–445. doi:10.1098/rspb.2001.1876. 11886634 PMC1690924

[17] Laven H 1967 Eradication of *Culex pipiens fatigans* through cytoplasmic incompatibility. Nature 216:383. doi:10.1038/216383a0. 4228275

[18] Lees RS, Gilles JR, Hendrichs J, Vreysen MJ, Bourtzis K 2015 Back to the future: the sterile insect technique against mosquito disease vectors. Curr Opin Insect Sci 10:156–162. doi:10.1016/j.cois.2015.05.011. 29588003

[19] O'Connor L, Plichart C, Sang AC, Brelsfoard CL, Bossin HC, Dobson SL 2012 Open release of male mosquitoes infected with a *Wolbachia* biopesticide: field performance and infection containment. PLoS Negl Trop Dis 6:e1797. doi:10.1371/journal.pntd.0001797. 23166845 PMC3499408

[20] Hoffmann AA, Montgomery BL, Popovici J, Iturbe-Ormaetxe I, Johnson PH, Muzzi F, Greenfield M, Durkan M, Leong YS, Dong Y, Cook H, Axford J, Callahan AG, Kenny N, Omodei C, McGraw EA, Ryan PA, Ritchie SA, Turelli M, O'Neill SL 2011 Successful establishment of *Wolbachia* in *Aedes* populations to suppress dengue transmission. Nature 476:454–457. doi:10.1038/nature10356. 21866160

[21] O’Neill SL 2018 The use of *Wolbachia* by the world mosquito program to interrupt transmission of *Aedes aegypti* transmitted viruses. Adv Exp Med Biol 1062:355–360. doi:10.1007/978-981-10-8727-1_24. 29845544

[22] O’Neill SL, Ryan PA, Turley AP, Wilson G, Retzki K, Iturbe-Ormaetxe I, Dong Y, Kenny N, Paton CJ, Ritchie SA 13 8 2018, posting date Scaled deployment of *Wolbachia* to protect the community from dengue and other *Aedes* transmitted arboviruses. Gates Open Res doi:10.12688/gatesopenres.12844.1. PMC6305154 30596205

[23] Blagrove MSC, Arias-Goeta C, Failloux A-B, Sinkins SP 2012 *Wolbachia* strain *w*Mel induces cytoplasmic incompatibility and blocks dengue transmission in *Aedes albopictus*. Proc Natl Acad Sci U S A 109:255–260. doi:10.1073/pnas.1112021108. 22123944 PMC3252941

[24] Dutra HLC, Dos Santos LMB, Caragata EP, Silva JBL, Villela DAM, Maciel-de-Freitas R, Moreira LA 2015 From lab to field: the influence of urban landscapes on the invasive potential of *Wolbachia* in Brazilian *Aedes aegypti* mosquitoes. PLoS Negl Trop Dis 9:e0003689. doi:10.1371/journal.pntd.0003689. 25905888 PMC4408005

[25] Bourtzis K, Nirgianaki A, Markakis G, Savakis C 1996 *Wolbachia* infection and cytoplasmic incompatibility in *Drosophila* species. Genetics 144:1063–1073. 8913750 10.1093/genetics/144.3.1063 PMC1207602

[26] Hoffmann AA, Clancy DJ, Merton E 1994 Cytoplasmic incompatibility in Australian populations of *Drosophila melanogaster*. Genetics 136:993–999. 8005448 10.1093/genetics/136.3.993 PMC1205902

[27] Hoffmann AA, Hercus M, Dagher H 1998 Population dynamics of the *Wolbachia* infection causing cytoplasmic incompatibility in *Drosophila melanogaster*. Genetics 148:221–231. 9475734 10.1093/genetics/148.1.221 PMC1459765

[28] Holden PR, Jones P, Brookfield JFY 1993 Evidence for a *Wolbachia* symbiont in *Drosophila melanogaster*. Genet Res 62:23. doi:10.1017/s0016672300031529. 7691685

[29] LePage DP, Metcalf JA, Bordenstein SR, On J, Perlmutter JI, Shropshire JD, Layton EM, Funkhouser-Jones LJ, Beckmann JF, Bordenstein SR 2017 Prophage WO genes recapitulate and enhance *Wolbachia*-induced cytoplasmic incompatibility. Nature 543:243–247. doi:10.1038/nature21391. 28241146 PMC5358093

[30] Reynolds KT, Hoffmann AA 2002 Male age, host effects and the weak expression or non-expression of cytoplasmic incompatibility in *Drosophila* strains infected by maternally transmitted *Wolbachia*. Genet Res 80:79–87. doi:10.1017/S0016672302005827. 12534211

[31] Solignac M, Vautrin D, Rousset F 1994 Widespread occurrence of the proteobacteria *Wolbachia* and partial cytoplasmic incompatibility in *Drosophila melanogaster*. C R Acad Sci III 317:461–470.

[32] Yamada R, Floate KD, Riegler M, O'Neill SL 2007 Male development time influences the strength of *Wolbachia*-induced cytoplasmic incompatibility expression in *Drosophila melanogaster*. Genetics 177:801–808. doi:10.1534/genetics.106.068486. 17660578 PMC2034644

[33] Clark ME, Veneti Z, Bourtzis K, Karr TL 2003 *Wolbachia* distribution and cytoplasmic incompatibility during sperm development: the cyst as the basic cellular unit of CI expression. Mech Dev 120:185–198. doi:10.1016/S0925-4773(02)00424-0. 12559491

[34] Shropshire JD, On J, Layton EM, Zhou H, Bordenstein SR 2018 One prophage WO gene rescues cytoplasmic incompatibility in *Drosophila melanogaster*. Proc Natl Acad Sci U S A 115:4987–4991. doi:10.1073/pnas.1800650115. 29686091 PMC5948995

[35] Crespigny FECD, Pitt TD, Wedell N 2006 Increased male mating rate in *Drosophila* is associated with *Wolbachia* infection. J Evol Biol 19:1964–1972. doi:10.1111/j.1420-9101.2006.01143.x. 17040394

[36] Boyle L, O'Neill SL, Robertson HM, Karr TL 1993 Interspecific and intraspecific horizontal transfer of *Wolbachia* in *Drosophila*. Science 260:1796–1799. doi:10.1126/science.8511587. 8511587

[37] Clark ME, Veneti Z, Bourtzis K, Karr TL 2002 The distribution and proliferation of the intracellular bacteria *Wolbachia* during spermatogenesis in *Drosophila*. Mech Dev 111:3–15. doi:10.1016/S0925-4773(01)00594-9. 11804774

[38] Veneti Z, Clark ME, Karr TL, Savakis C, Bourtzis K 2004 Heads or tails: host-parasite interactions in the *Drosophila*-*Wolbachia* system. Appl Environ Microbiol 70:5366–5372. doi:10.1128/AEM.70.9.5366-5372.2004. 15345422 PMC520876

[39] Flores HA, O'Neill SL 2018 Controlling vector-borne diseases by releasing modified mosquitoes. Nat Rev Microbiol 16:508–518. doi:10.1038/s41579-018-0025-0. 29777177 PMC7612058

[40] Christensen S, Camacho M, Sharmin Z, Momtaz AJMZ, Perez L, Navarro G, Triana J, Samarah H, Turelli M, Serbus L 2019 Quantitative methods for assessing local and bodywide contributions to *Wolbachia* titer in maternal germline cells of *Drosophila*. BMC Microbiol 19:206. doi:10.1186/s12866-019-1579-3. 31481018 PMC6724367

[41] Calvitti M, Marini F, Desiderio A, Puggioli A, Moretti R 2015 *Wolbachia* density and cytoplasmic incompatibility in *Aedes albopictus*: concerns with using artificial *Wolbachia* infection as a vector suppression tool. PLoS One 10:e0121813. doi:10.1371/journal.pone.0121813. 25812130 PMC4374832

[42] Tortosa P, Charlat S, Labbé P, Dehecq J-S, Barré H, Weill M 2010 *Wolbachia* sge-sex-specific density in *Aedes albopictus*: a host evolutionary response to cytoplasmic incompatibility? PLoS One 5:e9700. doi:10.1371/journal.pone.0009700. 20300514 PMC2838780

[43] Genty L-M, Bouchon D, Raimond M, Bertaux J 2014 *Wolbachia* infect ovaries in the course of their maturation: last minute passengers and priority travellers? PLoS One 9:e94577. doi:10.1371/journal.pone.0094577. 24722673 PMC3983217

[44] Guo Y, Hoffmann AA, Xu X-Q, Mo P-W, Huang H-J, Gong J-T, Ju J-F, Hong X-Y 28 8 2018, posting date Vertical transmission of Wolbachia is associated with host vitellogenin in *Laodelphax striatellus*. Front Microbiol doi:10.3389/fmicb.2018.02016. PMC6127624 30233514

[45] Fast EM, Toomey ME, Panaram K, Desjardins D, Kolaczyk ED, Frydman HM 2011 *Wolbachia* enhance *Drosophila* stem cell proliferation and target the germline stem cell niche. Science 334:990–992. doi:10.1126/science.1209609. 22021671 PMC4030408

[46] Frydman HM, Li JM, Robson DN, Wieschaus E 2006 Somatic stem cell niche tropism in *Wolbachia*. Nature 441:509–512. doi:10.1038/nature04756. 16724067

[47] Sacchi L, Genchi M, Clementi E, Negri I, Alma A, Ohler S, Sassera D, Bourtzis K, Bandi C 2010 Bacteriocyte-like cells harbour *Wolbachia* in the ovary of *Drosophila melanogaster* (Insecta, Diptera) and *Zyginidia pullula* (Insecta, Hemiptera). Tissue Cell 42:328–333. doi:10.1016/j.tice.2010.07.009. 20817243

[48] Toomey ME, Panaram K, Fast EM, Beatty C, Frydman HM 2013 Evolutionarily conserved *Wolbachia*-encoded factors control pattern of stem-cell niche tropism in *Drosophila* ovaries and favor infection. Proc Natl Acad Sci U S A 110:10788–10793. doi:10.1073/pnas.1301524110. 23744038 PMC3696799

[49] Deng W, Lin H 1997 Spectrosomes and fusomes anchor mitotic spindles during asymmetric germ cell divisions and facilitate the formation of a polarized microtubule array for oocyte specification in *Drosophila*. Dev Biol 189:79–94. doi:10.1006/dbio.1997.8669. 9281339

[50] Lin H, Schagat T 1997 Neuroblasts: a model for the asymmetric division of stem cells. Trends Genet 13:33–39. doi:10.1016/s0168-9525(96)10050-0. 9009846

[51] Presgraves DC 2000 A genetic test of the mechanism of *Wolbachia*-induced cytoplasmic incompatibility in *Drosophila*. Genetics 154:771–776. 10655228 10.1093/genetics/154.2.771 PMC1460966

[52] Shropshire JD, Bordenstein SR 2019 Two-by-one model of cytoplasmic incompatibility: synthetic recapitulation by transgenic expression of *cifA* and *cifB* in *Drosophila*. PLoS Genet 15:e1008221. doi:10.1371/journal.pgen.1008221. 31242186 PMC6594578

[53] Shropshire JD, Leigh B, Bordenstein SR, Duplouy A, Riegler M, Brownlie JC, Bordenstein SR 2019 Models and nomenclature for cytoplasmic incompatibility: caution over premature conclusions – a response to Beckmann et al. Trends Genet 35:397–399. doi:10.1016/j.tig.2019.03.004. 31003827

[54] Tram U, Sullivan W 2002 Role of delayed nuclear envelope breakdown and mitosis in *Wolbachia*-induced cytoplasmic incompatibility. Science 296:1124–1126. doi:10.1126/science.1070536. 12004132

[55] Ferree PM, Sullivan W 2006 A genetic test of the role of the maternal pronucleus in *Wolbachia*-induced cytoplasmic incompatibility in *Drosophila melanogaster*. Genetics 173:839–847. doi:10.1534/genetics.105.053272. 16624919 PMC1526499

[56] Poinsot D, Charlat S, Mercot H 2003 On the mechanism of *Wolbachia*-induced cytoplasmic incompatibility: confronting the models with the facts. Bioessays 25:259–265. doi:10.1002/bies.10234. 12596230

[57] Bossan B, Koehncke A, Hammerstein P 2011 A new model and method for understanding *Wolbachia*-induced cytoplasmic incompatibility. PLoS One 6:e19757. doi:10.1371/journal.pone.0019757. 21572955 PMC3091874

[58] Landmann F, Orsi GA, Loppin B, Sullivan W 2009 *Wolbachia*-mediated cytoplasmic incompatibility is associated with impaired histone deposition in the male pronucleus. PLoS Pathog 5:e1000343. doi:10.1371/journal.ppat.1000343. 19300496 PMC2652114

[59] Beckmann JF, Bonneau M, Chen H, Hochstrasser M, Poinsot D, Mercot H, Weill M, Sicard M, Charlat S 2019 The toxin-antidote model of cytoplasmic incompatibility: genetics and evolutionary implications. Trends Genet 35:175–185. doi:10.1016/j.tig.2018.12.004. 30685209 PMC6519454

[60] Beckmann JF, Ronau J, Hochstrasser M 2017 A *Wolbachia* deubiquitylating enzyme induces cytoplasmic incompatibility. Nat Microbiol 2:17007. doi:10.1038/nmicrobiol.2017.7. 28248294 PMC5336136

[61] Bressac C, Rousset F 1993 The reproductive incompatibility system in *Drosophila simulans*: DAPI-staining analysis of the *Wolbachia* symbionts in sperm cysts. J Invertebr Pathol 61:226–230. doi:10.1006/jipa.1993.1044. 7689622

[62] Snook RR, Cleland SY, Wolfner MF, Karr TL 2000 Offsetting effects of *Wolbachia* infection and heat shock on sperm production in *Drosophila simulans*: analyses of fecundity, fertility and accessory gland proteins. Genetics 155:167–178. 10790392 10.1093/genetics/155.1.167 PMC1461085

[63] Lindsley DL, Tokuyasu KT 1980 Spermatogenesis, p 226–287. *In* Ashburner M, Wright TRF (ed), Genetics and biology of Drosophila (2nd ed). Academic Press, San Diego, CA.

[64] ScienceDaily. 1 5 2018 Mechanisms for continually producing sperm. https://www.sciencedaily.com/releases/2015/05/150501095957.htm. Accessed 12 July 2019.

[65] Ruhmann H, Wensing KU, Neuhalfen N, Specker JH, Fricke C 2016 Early reproductive success in *Drosophila* males is dependent on maturity of the accessory gland. Behav Ecol 27:1859–1868.

[66] Karr TL, Yang W, Feder ME 1998 Overcoming cytoplasmic incompatibility in *Drosophila*. Proc Biol Sci 265:391–395. doi:10.1098/rspb.1998.0307. 9523438 PMC1688895

[67] Giardina TJ, Clark AG, Fiumera AC 2017 Estimating mating rates in wild *Drosophila melanogaster* females by decay rates of male reproductive proteins in their reproductive tracts. Mol Ecol Resour 17:1202–1209. doi:10.1111/1755-0998.12661. 28213940

[68] Chafee ME, Zecher CN, Gourley ML, Schmidt VT, Chen JH, Bordenstein SR, Clark ME, Bordenstein SR 2011 Decoupling of host-symbiont–phage coadaptations following transfer between insect species. Genetics 187:203–215. doi:10.1534/genetics.110.120675. 20944019 PMC3018312

[69] Miller WJ, Ehrman L, Schneider D 2010 Infectious speciation revisited: impact of symbiont-depletion on female fitness and mating behavior of *Drosophila paulistorum*. PLoS Pathog 6:e1001214. doi:10.1371/journal.ppat.1001214. 21151959 PMC2996333

[70] Vala F, Egas M, Breeuwer JAJ, Sabelis MW 2004 *Wolbachia* affects oviposition and mating behaviour of its spider mite host: *Wolbachia* induces assortative mating. J Evol Biol 17:692–700. doi:10.1046/j.1420-9101.2003.00679.x. 15149411

[71] Jaenike J, Dyer KA, Cornish C, Minhas MS 2006 Asymmetrical reinforcement and *Wolbachia* infection in *Drosophila*. PLoS Biol 4:e325. doi:10.1371/journal.pbio.0040325. 17032063 PMC1592313

[72] Ross PA, Ritchie SA, Axford JK, Hoffmann AA 2019 Loss of cytoplasmic incompatibility in *Wolbachia*-infected *Aedes aegypti* under field conditions. PLoS Negl Trop Dis 13:e0007357. doi:10.1371/journal.pntd.0007357. 31002720 PMC6493766

[73] Ritchie SA, van den Hurk AF, Smout MJ, Staunton KM, Hoffmann AA 2018 Mission accomplished? We need a guide to the ‘post release’ world of *Wolbachia* for *Aedes*-borne disease control. Trends Parasitol 34:217–226. doi:10.1016/j.pt.2017.11.011. 29396201

[74] Hoffmann AA, Turelli M, Simmons GM 1986 Unidirectional incompatibility between populations of *Drosophila simulans*. Evolution 40:692–701. doi:10.1111/j.1558-5646.1986.tb00531.x. 28556160

[75] Cooper BS, Ginsberg PS, Turelli M, Matute DR 2017 *Wolbachia* in the *Drosophila yakuba* complex: pervasive frequency variation and weak cytoplasmic incompatibility, but no apparent effect on reproductive isolation. Genetics 205:333–351. doi:10.1534/genetics.116.196238. 27821433 PMC5223512

[76] Charlat S, Ballard JWO, Mercot H 2004 What maintains noncytoplasmic incompatibility inducing *Wolbachia* in their hosts: a case study from a natural *Drosophila yakuba* population. J Evol Biol 17:322–330. doi:10.1046/j.1420-9101.2003.00676.x. 15009266

[77] Zabalou S, Charlat S, Nirgianaki A, Lachaise D, Mercot H, Bourtzis K 2004 Natural *Wolbachia* infections in the *Drosophila yakuba* species complex do not induce cytoplasmic incompatibility but fully rescue the *w*Ri modification. Genetics 167:827–834. doi:10.1534/genetics.103.015990. 15238531 PMC1470911

[78] Martinez J, Longdon B, Bauer S, Chan Y-S, Miller WK, Bourtzis K, Teixeira L, Jiggins FM 2014 Symbionts commonly provide broad spectrum resistance to viruses in insects: a comparative analysis of *Wolbachia* strains. PLoS Pathog 10:e1004369. doi:10.1371/journal.ppat.1004369. 25233341 PMC4169468

[79] Poinsot D, Bourtzis K, Markakis G, Savakis C, Mercot H 1998 *Wolbachia* transfer from *Drosophila melanogaster* into *D. simulans*: host effect and cytoplasmic incompatibility relationships. Genetics 150:227–237. 9725842 10.1093/genetics/150.1.227 PMC1460311

[80] Dyer KA, Minhas MS, Jaenike J 2005 Expression and modulation of embryonic male-killing in *Drosophila innubila*: opportunities for multilevel selection. Evol Int J Org Evol 59:838–848. doi:10.1111/j.0014-3820.2005.tb01757.x. 15926693

[81] Miller PB, Obrik-Uloho OT, Phan MH, Medrano CL, Renier JS, Thayer JL, Wiessner G, Bloch Qazi MC 2014 The song of the old mother: reproductive senescence in female *Drosophila*. Fly (Austin) 8:127–139. doi:10.4161/19336934.2014.969144. 25523082 PMC4594540

