# Supplementary material for: Paternal Grandmother Age Affects the Strength of Wolbachia-Induced Cytoplasmic Incompatibility in Drosophila melanogaster
Source: mBio. 2019 Nov 5;10(6):e01879-19. doi: 10.1128/mBio.01879-19 (PMC6831774; doi:10.1128/mBio.01879-19)
Supplement: TABLE S1 [file mBio.01879-19-st001.docx]

**Table S1. P-values associated with all statistical comparisons made in main and extended data figures.**

| Figure | Comparison | *P* value | Test |
| --- | --- | --- | --- |
| Fig. 1 | All CI crosses | **<0.0001** | Kruskal-Wallis |
|  | [2d.Mo;M;+]x[F;-] vs. [5d.Mo;M;+]x[F;-] | >0.9999 | Kruskal-Wallis with Dunn's multiple comparisons |
|  | [2d.Mo;M;+]x[F;-] vs. [11d.Mo;M;+]x[F;-] | 0.1279 | Kruskal-Wallis with Dunn's multiple comparisons |
|  | [2d.Mo;M;+]x[F;-] vs. [14d.Mo;M;+]x[F;-] | **0.0031** | Kruskal-Wallis with Dunn's multiple comparisons |
|  | [2d.Mo;M;+]x[F;-] vs. [18d.Mo;M;+]x[F;-] | **0.0005** | Kruskal-Wallis with Dunn's multiple comparisons |
|  | [5d.Mo;M;+]x[F;-] vs. [11d.Mo;M;+]x[F;-] | 0.2238 | Kruskal-Wallis with Dunn's multiple comparisons |
|  | [5d.Mo;M;+]x[F;-] vs. [14d.Mo;M;+]x[F;-] | **0.0095** | Kruskal-Wallis with Dunn's multiple comparisons |
|  | [5d.Mo;M;+]x[F;-] vs. [18d.Mo;M;+]x[F;-] | **0.0018** | Kruskal-Wallis with Dunn's multiple comparisons |
|  | [11d.Mo;M;+]x[F;-] vs. [14d.Mo;M;+]x[F;-] | >0.9999 | Kruskal-Wallis with Dunn's multiple comparisons |
|  | [11d.Mo;M;+]x[F;-] vs. [18d.Mo;M;+]x[F;-] | >0.9999 | Kruskal-Wallis with Dunn's multiple comparisons |
|  | [14d.Mo;M;+]x[F;-] vs. [18d.Mo;M;+]x[F;-] | >0.9999 | Kruskal-Wallis with Dunn's multiple comparisons |
| Fig. 2A | All groups | 0.0218 | Kruskal-Wallis |
|  | [2d.Mo;M;+;v;ab] vs. [5d.Mo;M;+;v;ab] | >0.9999 | Kruskal-Wallis with Dunn's multiple comparisons |
|  | [2d.Mo;M;+;v;ab] vs. [11d.Mo;M;+;v;ab] | >0.9999 | Kruskal-Wallis with Dunn's multiple comparisons |
|  | [2d.Mo;M;+;v;ab] vs. [14d.Mo;M;+;v;ab] | >0.9999 | Kruskal-Wallis with Dunn's multiple comparisons |
|  | [2d.Mo;M;+;v;ab] vs. [18d.Mo;M;+;v;ab] | **0.045** | Kruskal-Wallis with Dunn's multiple comparisons |
|  | [5d.Mo;M;+;v;ab] vs. [11d.Mo;M;+;v;ab] | >0.9999 | Kruskal-Wallis with Dunn's multiple comparisons |
|  | [5d.Mo;M;+;v;ab] vs. [14d.Mo;M;+;v;ab] | >0.9999 | Kruskal-Wallis with Dunn's multiple comparisons |
|  | [5d.Mo;M;+;v;ab] vs. [18d.Mo;M;+;v;ab] | 0.052 | Kruskal-Wallis with Dunn's multiple comparisons |
|  | [11d.Mo;M;+;v;ab] vs. [14d.Mo;M;+;v;ab] | >0.9999 | Kruskal-Wallis with Dunn's multiple comparisons |
|  | [11d.Mo;M;+;v;ab] vs. [18d.Mo;M;+;v;ab] | >0.9999 | Kruskal-Wallis with Dunn's multiple comparisons |
|  | [14d.Mo;M;+;v;ab] vs. [18d.Mo;M;+;v;ab] | >0.9999 | Kruskal-Wallis with Dunn's multiple comparisons |
| Fig. 2B | [2d.Mo;+;emb] vs. [11d.Mo;+;emb] | **0.0006** | Mann-Whitney *U* |
| Fig. 3 | All groups | **<0.0001** | Kruskal-Wallis |
|  | [2d.;F;+;v;ov] vs. [11d.;F;+;v;ov] | **0.0045** | Kruskal-Wallis with Dunn's multiple comparisons |
|  | [2d.;F;+;v;ov] vs. [2d.;F;+;nv;ov] | 0.0882 | Kruskal-Wallis with Dunn's multiple comparisons |
|  | [2d.;F;+;v;ov] vs. [11d.;F;+;nv;ov] | >0.9999 | Kruskal-Wallis with Dunn's multiple comparisons |
|  | [11d.;F;+;v;ov] vs. [2d.;F;+;nv;ov] | **<0.0001** | Kruskal-Wallis with Dunn's multiple comparisons |
|  | [11d.;F;+;v;ov] vs. [11d.;F;+;nv;ov] | **0.024** | Kruskal-Wallis with Dunn's multiple comparisons |
|  | [2d.;F;+;nv;ov] vs. [11d.;F;+;nv;ov] | **0.0087** | Kruskal-Wallis with Dunn's multiple comparisons |
| Fig. S1 | All CI crosses | **0.0006** | Kruskal-Wallis |
|  | All rescue crosses | 0.3705 | Kruskal-Wallis |
|  | [2d.Mo;M;+]x[F;-] vs. [5d.Mo;M;+]x[F;-] | 0.9624 | Kruskal-Wallis with Dunn's multiple comparisons |
|  | [2d.Mo;M;+]x[F;-] vs. [11d.Mo;M;+]x[F;-] | **0.0008** | Kruskal-Wallis with Dunn's multiple comparisons |
|  | [2d.Mo;M;+]x[F;-] vs. [14d.Mo;M;+]x[F;-] | **0.011** | Kruskal-Wallis with Dunn's multiple comparisons |
|  | [5d.Mo;M;+]x[F;-] vs. [11d.Mo;M;+]x[F;-] | 0.1606 | Kruskal-Wallis with Dunn's multiple comparisons |
|  | [5d.Mo;M;+]x[F;-] vs. [14d.Mo;M;+]x[F;-] | 0.7414 | Kruskal-Wallis with Dunn's multiple comparisons |
|  | [11d.Mo;M;+]x[F;-] vs. [14d.Mo;M;+]x[F;-] | >0.9999 | Kruskal-Wallis with Dunn's multiple comparisons |
|  | [2d.Mo;M;+]x[F;+] vs. [5d.Mo;M;+]x[F;+] | >0.9999 | Kruskal-Wallis with Dunn's multiple comparisons |
|  | [2d.Mo;M;+]x[F;+] vs. [11d.Mo;M;+]x[F;+] | >0.9999 | Kruskal-Wallis with Dunn's multiple comparisons |
|  | [2d.Mo;M;+]x[F;+] vs. [14d.Mo;M;+]x[F;+] | >0.9999 | Kruskal-Wallis with Dunn's multiple comparisons |
|  | [5d.Mo;M;+]x[F;+] vs. [11d.Mo;M;+]x[F;+] | >0.9999 | Kruskal-Wallis with Dunn's multiple comparisons |
|  | [5d.Mo;M;+]x[F;+] vs. [14d.Mo;M;+]x[F;+] | 0.6495 | Kruskal-Wallis with Dunn's multiple comparisons |
|  | [11d.Mo;M;+]x[F;+] vs. [14d.Mo;M;+]x[F;+] | >0.9999 | Kruskal-Wallis with Dunn's multiple comparisons |
| Fig. S2 | All uninfected crosses | 0.3907 | Kruskal-Wallis |
|  | All CI crosses | **0.0042** | Kruskal-Wallis |
|  | [2d.Mo;M;-]x[F;-] vs. [5d.Mo;M;-]x[F;-] | 0.6493 | Kruskal-Wallis with Dunn's multiple comparisons |
|  | [2d.Mo;M;-]x[F;-] vs. [11d.Mo;M;-]x[F;-] | >0.9999 | Kruskal-Wallis with Dunn's multiple comparisons |
|  | [5d.Mo;M;-]x[F;-] vs. [11d.Mo;M;-]x[F;-] | 0.8411 | Kruskal-Wallis with Dunn's multiple comparisons |
|  | [2d.Mo;M;+]x[F;-] vs. [5d.Mo;M;+]x[F;-] | >0.9999 | Kruskal-Wallis with Dunn's multiple comparisons |
|  | [2d.Mo;M;+]x[F;-] vs. [11d.Mo;M;+]x[F;-] | **0.0033** | Kruskal-Wallis with Dunn's multiple comparisons |
|  | [5d.Mo;M;+]x[F;-] vs. [11d.Mo;M;+]x[F;-] | 0.2917 | Kruskal-Wallis with Dunn's multiple comparisons |
| Fig. S3 | All virgin females | **<0.0001** | Kruskal-Wallis |
|  | All mated females | **<0.0001** | Kruskal-Wallis |
|  | [2d.;F;+;v;ov; groEL] vs. [2d.;F;+;v;ov; rp49] | 0.3476 | Kruskal-Wallis with Dunn's multiple comparisons |
|  | [2d.;F;+;v;ov; groEL] vs. [11d.;F;+;v;ov; groEL] | **<0.0001** | Kruskal-Wallis with Dunn's multiple comparisons |
|  | [2d.;F;+;v;ov; groEL] vs. [11d.;F;+;v;ov; rp49] | 0.0758 | Kruskal-Wallis with Dunn's multiple comparisons |
|  | [2d.;F;+;v;ov; rp49] vs. [11d.;F;+;v;ov; groEL] | **0.0131** | Kruskal-Wallis with Dunn's multiple comparisons |
|  | [2d.;F;+;v;ov; rp49] vs. [11d.;F;+;v;ov; groEl] | >0.9999 | Kruskal-Wallis with Dunn's multiple comparisons |
|  | [11d.;F;+;v;ov; groEL] vs. [11d.;F;+;v;ov; rp49] | 0.0605 | Kruskal-Wallis with Dunn's multiple comparisons |
|  | [2d.;F;+;nv;ov; groEL] vs. [2d.;F;+;nv;ov; rp49] | **0.0004** | Kruskal-Wallis with Dunn's multiple comparisons |
|  | [2d.;F;+;nv;ov; groEL] vs. [11d.;F;+;nv;ov; groEL] | **0.0102** | Kruskal-Wallis with Dunn's multiple comparisons |
|  | [2d.;F;+;nv;ov; groEL] vs. [11d.;F;+;nv;ov; rp49] | **<0.0001** | Kruskal-Wallis with Dunn's multiple comparisons |
|  | [2d.;F;+;nv;ov; rp49] vs. [11d.;F;+;nv;ov; groEL] | >0.9999 | Kruskal-Wallis with Dunn's multiple comparisons |
|  | [2d.;F;+;nv;ov; rp49] vs. [11d.;F;+;nv;ov; rp49] | >0.9999 | Kruskal-Wallis with Dunn's multiple comparisons |
|  | [11d.;F;+;nv;ov; groEL] vs. [11d.;F;+;nv;ov; rp49] | 0.7863 | Kruskal-Wallis with Dunn's multiple comparisons |
| Fig. S4 | All groups | **<0.0001** | Kruskal-Wallis |
|  | [2d.;F;+;v;ab] vs. [11d.;F;+;v;ab] | **<0.0001** | Kruskal-Wallis with Dunn's multiple comparisons |
|  | [2d.;F;+;v;ab] vs. [2d.;F;+;nv;ab] | >0.9999 | Kruskal-Wallis with Dunn's multiple comparisons |
|  | [2d.;F;+;v;ab] vs. [11d.;F;+;nv;ab] | 0.6563 | Kruskal-Wallis with Dunn's multiple comparisons |
|  | [11d.;F;+;v;ab] vs. [2d.;F;+;nv;ab] | **0.0094** | Kruskal-Wallis with Dunn's multiple comparisons |
|  | [11d.;F;+;v;ab] vs. [11d.;F;+;nv;ab] | 0.2291 | Kruskal-Wallis with Dunn's multiple comparisons |
|  | [2d.;F;+;nv;ab] vs. [11d.;F;+;nv;ab] | >0.9999 | Kruskal-Wallis with Dunn's multiple comparisons |
| #d.Mo = mother age, #d. = age, M = male, F = female, + = Wolbachia infected, - = Wolbachia uninfected, v = virgin, nv = not virgin, ab = abdomen, emb = embryo, ov = ovaries, bold *P* values = significant | | | |
