# Supplementary figures and images for: Paternal Grandmother Age Affects the Strength of Wolbachia-Induced Cytoplasmic Incompatibility in Drosophila melanogaster
Source: mBio. 2019 Nov 5;10(6):e01879-19. doi: 10.1128/mBio.01879-19 (PMC6831774; doi:10.1128/mBio.01879-19)

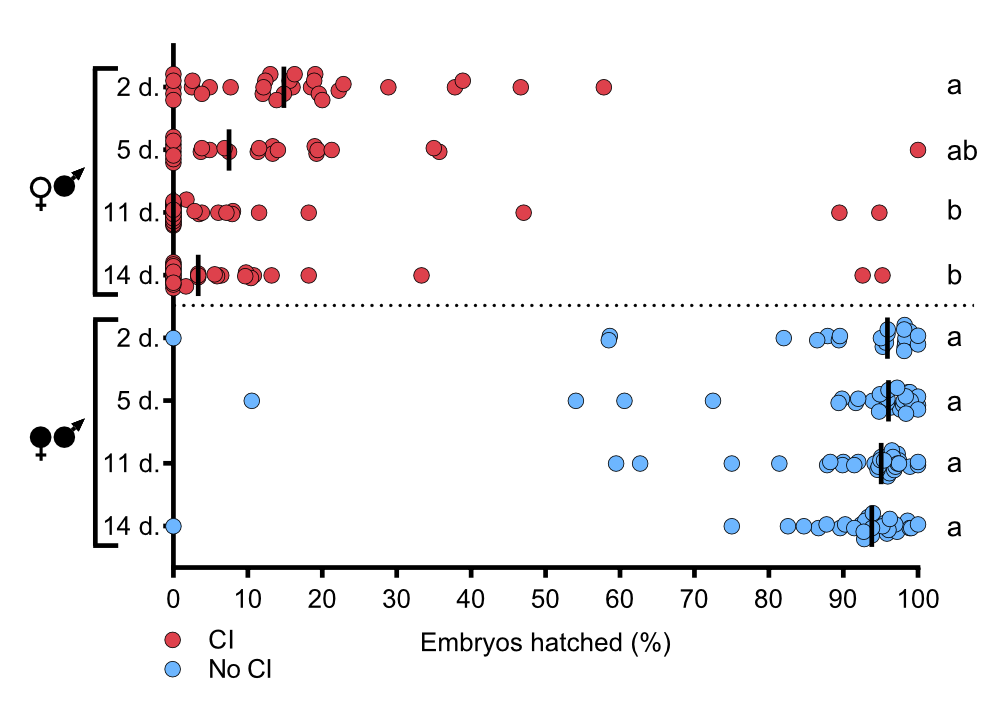

Supplement: FIG S1 [file mBio.01879-19-sf001.tif]

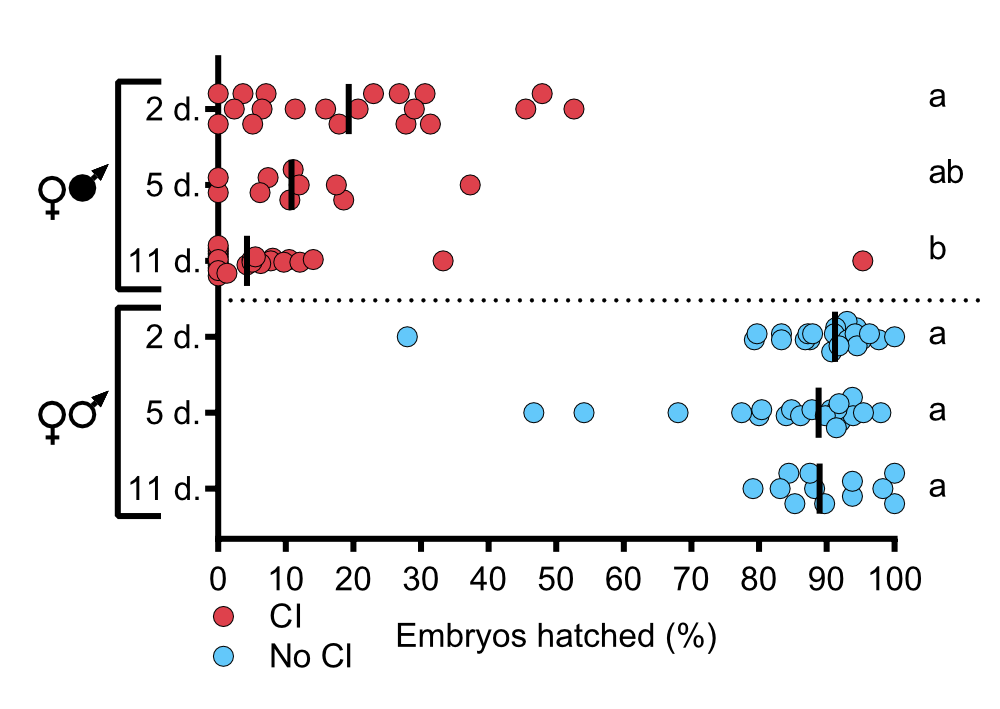

Supplement: FIG S2 [file mBio.01879-19-sf002.tif]

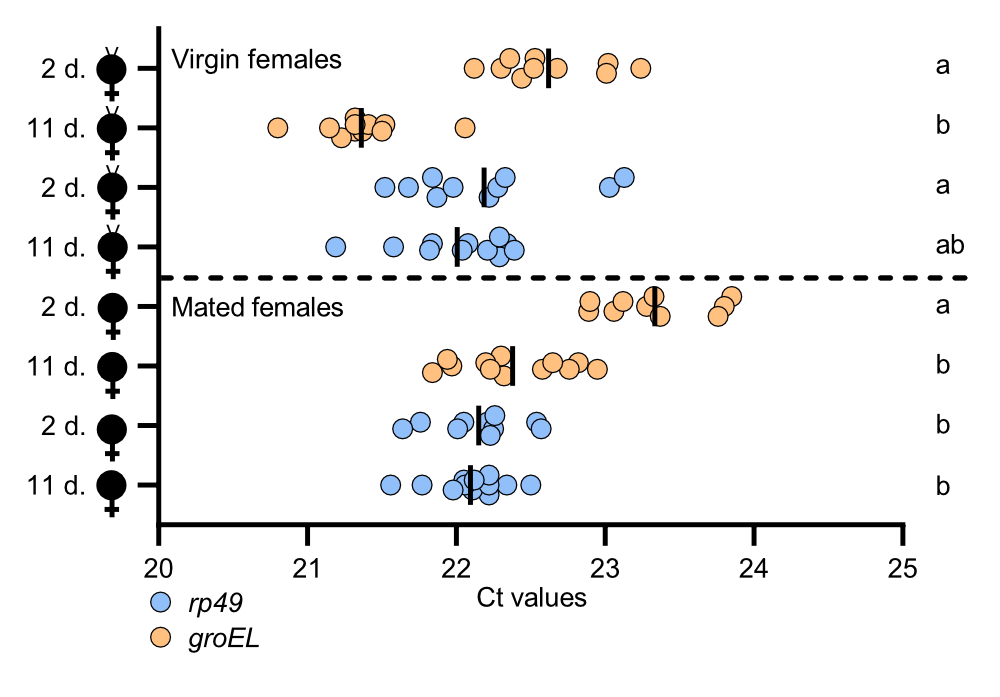

Supplement: FIG S3 [file mBio.01879-19-sf003.tif]

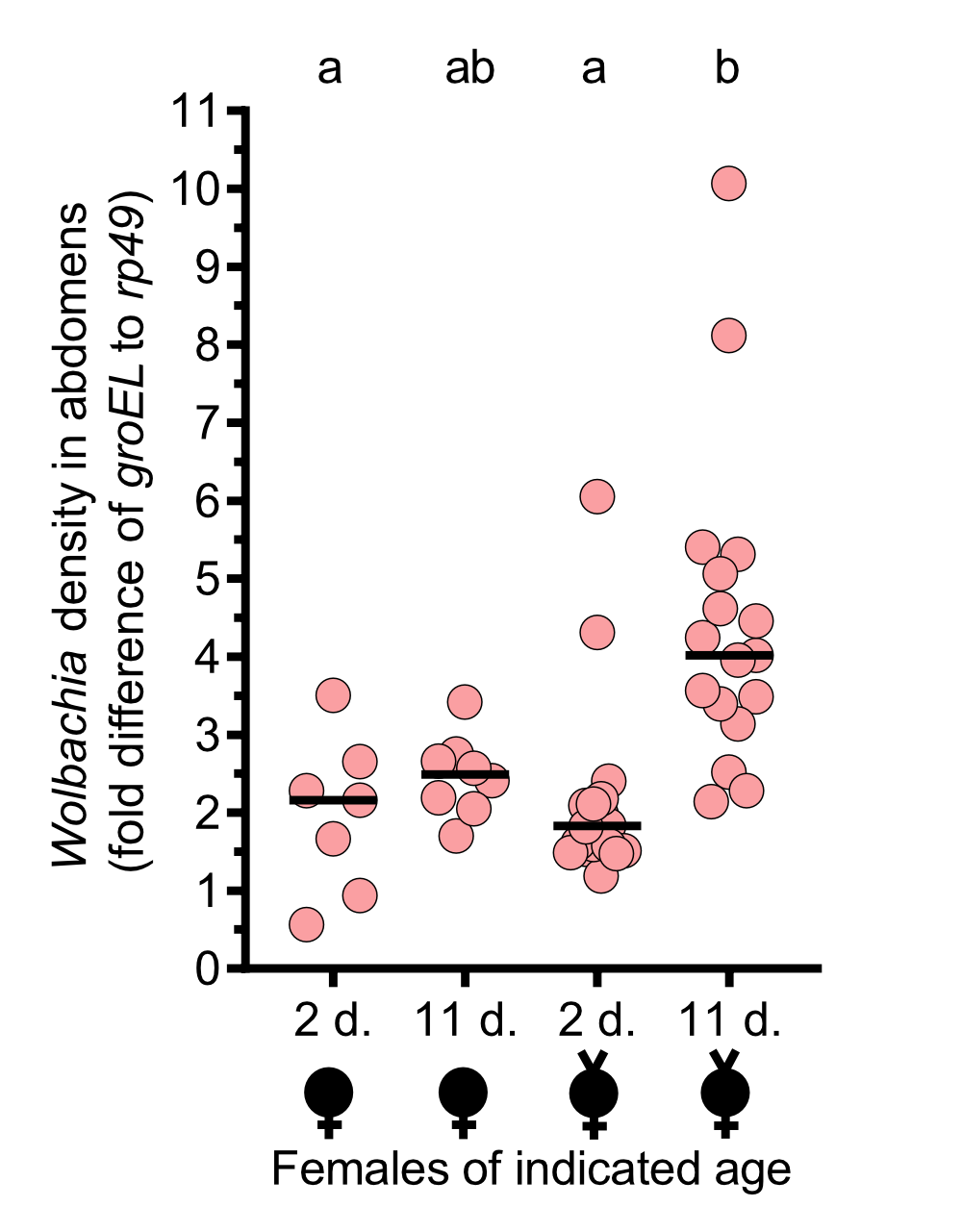

Supplement: FIG S4 [file mBio.01879-19-sf004.tif]
