# Supplementary material for: Paternal Grandmother Age Affects the Strength of Wolbachia-Induced Cytoplasmic Incompatibility in Drosophila melanogaster
Source: mBio. 2019 Nov 5;10(6):e01879-19. doi: 10.1128/mBio.01879-19 (PMC6831774; doi:10.1128/mBio.01879-19)
Supplement: TABLE S2 [file mBio.01879-19-st002.docx]

**Table S2. Primers used for qPCR.**

| **Primer** | **Sequence** | **Product Length (bp)** | **Source** |
| --- | --- | --- | --- |
| **Rp49_F** | CGGTTACGGATCGAACAAGC | **154** | [29] |
| **Rp49_R** | CTTGCGCTTCTTGGAGGAGA |  |  |
| **groEL_F** | CTAAAGTGCTTAATGCTTCACCTTC | **97** | [29] |
| **groEL_R** | CAACCTTTACTTCCTATTCTTG |  |  |
| F = forward primer, R = reverse primer | | | |
